# Serum and Saliva Level of miR-31-5p and miR-let 7a in EBV Associated Oropharyngeal Cancer

**DOI:** 10.3390/ijms241511965

**Published:** 2023-07-26

**Authors:** Anna Polz, Kamal Morshed, Robert Bibik, Bartłomiej Drop, Andrzej Drop, Małgorzata Polz-Dacewicz

**Affiliations:** 1 Synevo Poland, 80-180 Gdańsk, Poland; anna.polz@synevo.pl; 2 Department of Otolaryngology Head and Neck Cancer, University of Technology and Humanities in Radom, 26-600 Radom, Poland; k.morshed@uthrad.pl; 3 Department of Radiation Oncology, Oncology Center of Radom, 26-600 Radom, Poland; r.bibik@onkologiaradom.pl; 4 Department of Computer Science and Medical Statistics with the e-Health Laboratory, 20-090 Lublin, Poland; bartlomiej.drop@umlub.pl; 5 1st Department of Medical Radiology, Medical University of Lublin, 20-093 Lublin, Poland; 6 Department of Virology with Viral Diagnostics Laboratory, Medical University of Lublin, 20-093 Lublin, Poland

**Keywords:** oropharyngeal cancer, EBV, miR-31-5p, miR-let 7a

## Abstract

Epstein-Barr virus (EBV) has a well-documented association with head and neck neoplasms, including nasopharyngeal carcinoma (NPC). In the last few years, research aimed at elucidating the role of the miRs in the pathogenesis of head and neck cancer (HNC) has gained importance. The study of miRs expression has set new directions in the search for biomarkers with diagnostic and prognostic value, and even in the search for new therapeutic targets for various tumors, including HNC. The aim of current study was to approximate the importance of miR-31-5p and miR-let 7a in the pathogenesis of EBV associated oropharyngeal cancer. For this purpose, experiments were carried out to determine the level of mentioned miRs in serum among patients diagnosed with oropharyngeal cancer linked to EBV infection, depending on histological differentiation-grading (G1–G3) and TNM classification. All clinical specimens stratified by HPV status were HPV negative. The level of antibodies EBNA and EBVCA was also assessed. The obtained results showed a significantly increased serum level of miR-31-5p but decreased level of miR-let 7a in EBV positive oropharyngeal cancer patients. We demonstrated association between the level of tested miRs and clinical stage. Our findings showed that miR-31-5p and miR-let-7a may be involved in development and progression of EBV associated oropharyngeal cancer. Therefore, it seems important to further study these molecules, as well as to determine whether they could be important biomarkers in the diagnosis of oropharyngeal cancer associated with EBV infection.

## 1. Introduction

Head and neck cancers (HNCs) are very important problem of public health and are seventh the most common malignant tumors all over the world [1,2]. More than 660,000 new cases and 325,000 deaths are registered annually. A further gradual increase in both morbidity and mortality is expected [3]. In Poland, malignant tumors are the second cause of death after cardiovascular disease, where in 2019 alone, the Polish National Cancer Registry reported 171,000 new tumor cases and 100,000 deaths due to cancer [4]. However, about 5000 new HNC cases are registered every year [5].

Most HNCs arise from the mucosal epithelium of the oral cavity, pharynx, and larynx and are referred to as squamous cell carcinoma of the head and neck (HNSCC). The early stage of the disease is usually asymptomatic, and the cancer is detected late, increasing the mortality rate.

Many different factors, well-established in the literature, such as smoking, alcohol abuse, poor oral hygiene and dietary habits play a pivotal role in the development of HNC [6,7]. The multifactorial etiology of head and neck tumors includes also oncogenic viruses, i.e., Human papillomavirus (HPV) and Epstein–Barr virus (EBV) [8,9,10,11,12,13,14].

Oropharyngeal squamous cell carcinoma (OPSCC) is one of the cancers with a rapidly increasing incidence, especially associated with HPV infection (most common in men and lifetime number of sexual partners) [15]. OPCs have been dived into two distinct entities with different molecular profiles, tumor characteristics and prognosis, i.e., HPV positive and HPV negative [16,17,18]. Of all HPV-positive OPSCCs, 85–96% are due to HPV-16 infection. They will likely be preventable through prophylactic HPV vaccination, which is now being given to both boys and girls in several countries. Due to the significantly better prognosis of patients with HPV-associated OPSCC than HPV-negative OPSCC, the US Joint Committee on Cancer TNM 8 recommends stratification all cases by HPV status.

Targeted therapy is used in modern oncology. Despite clinical classification and pathological evaluation, accurate tools are needed to identify specific subgroups of patients. Therefore, new cancer biomarkers with a positive predictive value are constantly being sought [19].

Biomarkers are molecules present in tissues and body fluids, produced by cancer cells or normal cells in response to the ongoing cancer process. Recently, many researchers have focused on the detection of selected microRNAs (miRs) as potential diagnostic and prognostic biomarkers, as well as on the monitoring of changes in their profile [20,21,22,23].

MiRs are short, evolutionarily conserved, non-coding, single stranded RNA molecules (20–25 nucleotides) that regulate gene expression at the post-transcriptional level by binding to mRNA. They affect in number of biological processes such as differentiation, proliferation, inflammation, angiogenesis, migration and apoptosis [24,25]. These molecules are present in serum, saliva, plasma and other body fluids and also in organ tissue. Normal and neoplastic tissues can be distinguished by the level of miR expression. Profiling of miR expression can be a good diagnostic tool for assessing the severity of the disease or estimating survival time, and it can also be helpful in choosing personalized therapy [26].

Many studies investigate the expression of various miRs that may be important in the diagnosis and prognosis of oral cancer. There is growing scientific evidence showing that some miRs are expressed differently in oral cancer, which could help distinguish oral cancer patients from healthy individuals [26]. According to data from the literature, the expression of miR-21, -23a, -16, -29, -31, -34a, 375, -99, -200, -125a/b, -196a/b, -9, -181a, -155, -146a, and let-7 is most frequently studied [27,28]. Based on the results of the above- mentioned meta-analyses, two miRs were selected, i.e., miR-31-5p and miR-let 7a.

MiR-31-5p can act as an oncogene or tumor suppressor. The differential expression of this miR and its important role in oncogenesis has been reported in several cancers, making miR-31 a potential biomarker of these diseases [29,30,31].

The miR-let 7 family consists of 12 different members that are expressed in human [32]. In many different types of human cancers, the level of miR-let 7 is reduced what is most commonly associated with poor prognosis and probably may have therapeutic potential [33,34].

Almost all studies look at miR expression in the OSCC. However, there are no studies evaluating the level of miRs in cancers associated with EBV infection. Therefore, in the current study, we aimed to examine a potential involvement of EBV in the deregulation of miR 31-5p and miR-let 7a in oropharyngeal cancer patients and to determine whether these miRs could serve as diagnostic and/or prognostic non-invasive biomarkers.

For this purpose, the serum and saliva level of these miRs was analyzed in two groups of patients: EBV DNA positive and EBV DNA negative. The relationship between miRs level and TN classification and histological differentiation was also investigated.

Moreover, the level of antibodies against EBV antigens (anti-VCA in the IgM and IgG class and anti-EBNA in the IgG class) was assessed.

## 2. Results

The oropharyngeal cancer group of patients consisted of 42 subjects with EBV DNA detected in the tumor tissue, hereinafter referred as EBV positive—EBV(+) and 36 individuals without EBV DNA detected, hereinafter referred to as EBV negative EBV(−).

### 2.1. The Serum Level of miR and Anti-EBV Antibodies in Oropharyngeal Patients Compared to the Control Group

**Figure 1:**
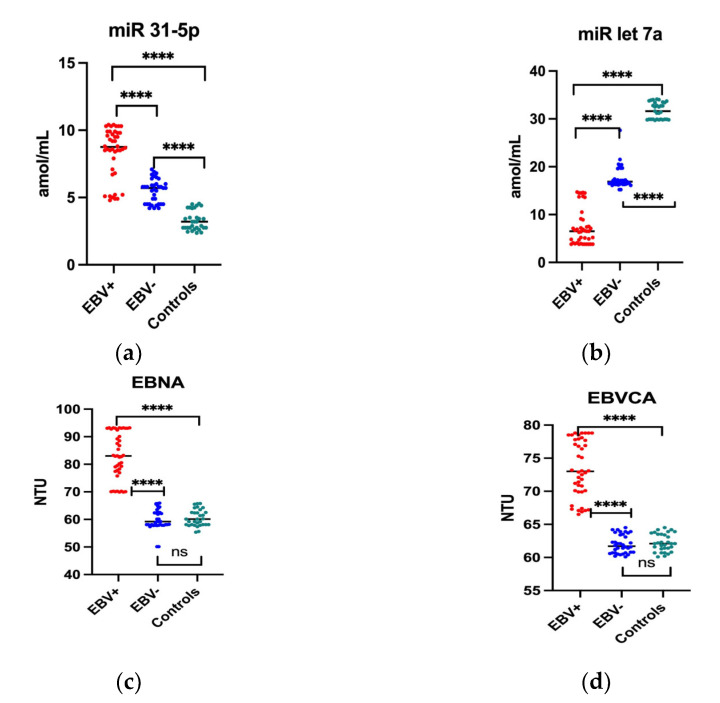
Serum level of studied parameters in EBV positive and EBV negative oropharyngeal patients in comparison to the control group. (**a**) miR 31-5p; (**b**) miR let 7a; (**c**) EBNA; (**d**) EBVCA. Mann Whitney U Test; Kruskal Wallis Test; **** *p* < 0.0001. miR 31-5p, -let 7a—amol/mL; EBNA and EBVCA—NTU (NovaTec Units). ns–not statistically significant; dots: red EBV+. Blue–EBV−, green–Controls.

In the first stage of the study, the level of miR 31-5p and miR-let 7a as well as anti-EBV antibodies, i.e., EBNA and EBVCA, was determined in the group of patients with oropharyngeal cancer EBV positive and EBV negative in comparison with the control group. The obtained results are presented in Figure 1. The miR-31-5p level was significantly higher in both the EBV+ and EBV− patients than in the control group (Figure 1a). Moreover, miR-31-5p level was significantly higher in EBV-positive oropharyngeal cancer patients than in EBV-negative patients (8.4 vs. 5.4 amol/mL; *p* < 0.0001)).

However, as the analysis showed, the level of miR-let 7a was significantly lower in the serum of patients with oropharyngeal cancer than in the control group. It has been observed significantly lower level of this molecule in the EBV+ group compared to the EBV− group, and was respectively 7.3 vs. 17.7 amol/mL(*p* < 0.0001) (Figure 1b).

As showed conducted analysis, the anti-VCA antibodies in the IgM class were not found both in the patients and in the control group. The serum level of EBNA in class IgG and EBVCA in class IgG antibodies of EBV-negative subjects and in the control group was similar. On the other hand, the level of both antibodies in EBV positive patients was significantly higher than in EBV negative group and was respectively: EBNA-83 NTU, EBVCA-73.4 NTU (*p* < 0.0001).

### 2.2. Saliva Level of Studied miRs

In recent years, scientific papers have been published whose authors examined the presence of various biomarkers in saliva. Saliva contains a wide spectrum of proteins, nucleic acids, electrolytes, hormones, enzymes, interleukin and miRNAs that can be used as non-invasive diagnostic and/or prognostic biomarkers. Due to this fact, we assessed the level of selected miRs in terms of their possible practical application for diagnostics oropharyngeal cancer linked to EBV.

**Figure 2:**
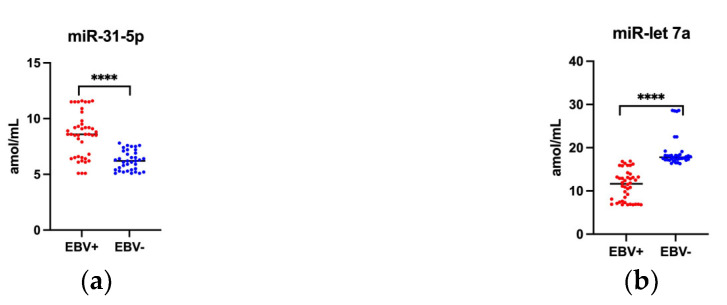
The level of tested miRs in saliva of oropharyngeal cancer patients: (**a**) miR-31-5p in EBV+ and in EBV−; (**b**) miR-let 7a in EBV+ and in EBV−. **** *p* < 0.0001.

The conducted studies showed that the saliva level of miR-31-5p was significantly higher in EBV positive than in EBV negative oropharyngeal cancer patients—8.5 vs. 6.2 amol/mL (Figure 2a). While the miR-let 7a levels were downregulated in EBV positive patients and was respectively 11.3 vs. 19.1 amol/mL (Figure 2b).

### 2.3. Serum Level of miR-31-5p and miR-let 7a by G T, N Classification among EBV Positive Oropharyngeal Cancer Patients

**Figure 3:**
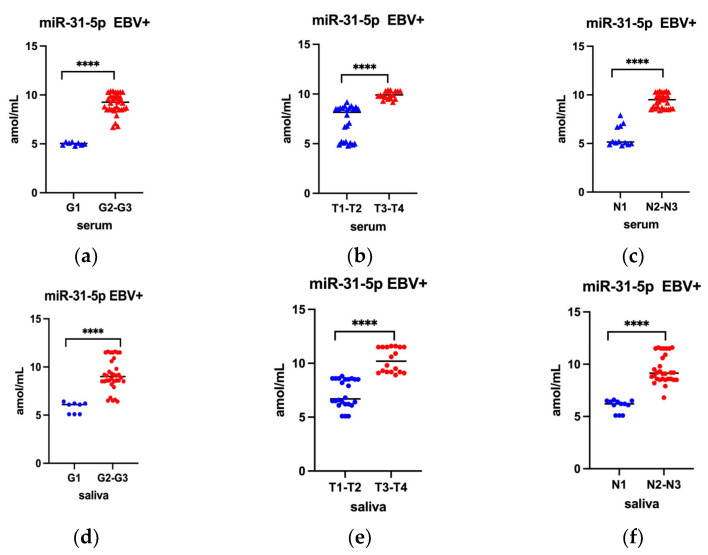
Serum level (**a**–**c**) of miR-31-5p by histological differentiation G (**a**), T (**b**) and N (**c**) classification; Saliva level (**d**–**f**) of miR-31-5p by G (**d**), T (**e**), and N (**f**) classification; Mann-Whitney U Test; **** *p* < 0.0001.

**Figure 4:**
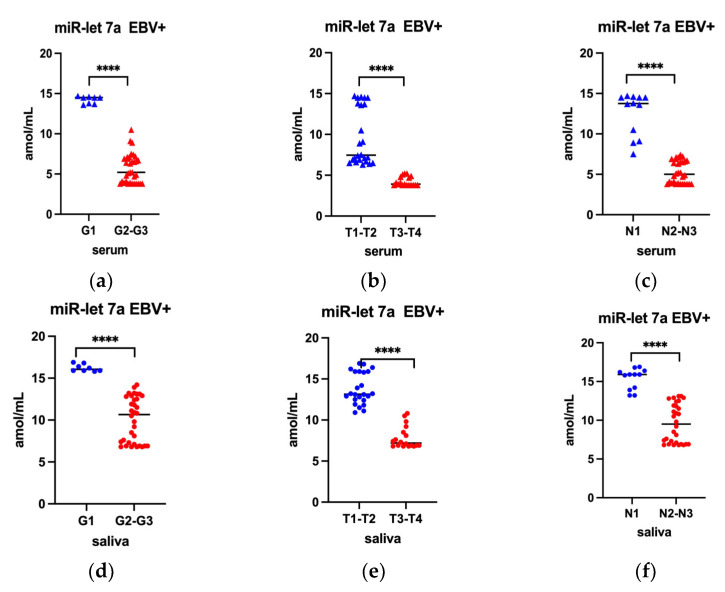
MiR-let 7a in serum (**a**–**c**) and in saliva (**d**–**f**) of EBV positive oropharyngeal cancer patients by GTN classification; Mann Whitney U Test; **** *p* < 0.0001.

In the next step of the presented research, the level of miR-31-5p and miR-let 7a were compared depending on the histological grading (G) as well as TN stage. In line with our assumptions, this analysis included only EBV positive cases. The results of this analysis are presented in Figure 3 (applies to miR-31-5p) and Figure 4 (applies to miR-let 7a).

Analysis of miR 31-5p level and grading, as well as TN stage, revealed that miR 31-5p level was statistically significant higher in moderately and poorly differentiated tumors (G2–G3) (Figure 3a), in extensive tumor dimensions (T3–T4) (Figure 3b) and in lesions with lymph nodes involvement (N2-N3) (Figure 3c). A similar relationship was observed when examining the level of miR- 31-5p in saliva (Figure 3d–f).

In turn, the analysis of the obtained results, taking into account the miR-let 7a level, showed a statistically significant decrease in the serum level in moderately and poorly differentiated tumor (Figure 4a), in extensive tumor dimensions (Figure 4b) and in lesions with lymph nodes involvement (Figure 4c). A similar trend was observed for miR-let 7a levels in saliva (Figure 4d–f).

## 3. Discussion

EBV establishes persistent infection in affected host cells and reactivates in the head and neck epithelium, influencing the pathogenesis of EBV-associated cancers [35,36]. However, the exact mechanism of this process is still not fully understood. In one of our team’s first studies on the prevalence of oncogenic viruses in oropharyngeal cancer, HPV was detected in 26.7% of cases and EBV in 53.3% of cases [37]. Due to the high percentage of detected EBV DNA, we decided to analyze these cases in various aspects. In the presented studies, an attempt was made to assess the impact of EBV infection on the level of selected miRs in oropharyngeal cancer. To the best of our knowledge, this study is the first original observation that implicates EBV infection with selected miRNA (miR-let 7a, miR-31-5p) in EBV associated oropharyngeal cancer in the Polish population.

As indicated by many studies conducted at the molecular level, genetic and epigenetic changes in cancer cells, as well as rearrangement of tumor microenvironment components (TME), affect the process of tumorigenesis.

TME may vary in different types of cancer but always consists of a cellular component containing tumor cells and stromal cells embedded in the extracellular matrix (ECM)—a non-cellular component containing collagen, fibronectin, hyaluronan, laminin. Interactions between all these elements rely on a complex network of cytokines, growth factors, inflammatory mediators and matrix remodeling enzymes and may promote the growth and invasion of cancer cells [38,39,40]. In addition, the interaction between host cells and viral agents can lead to the creation of a microenvironment conducive to oncogenesis [41].

TME is a complex, heterogeneous and constantly modified ecosystem. MiRs play an important role in remodeling of TME, hence the great interest in miRs as markers useful both from a diagnostic and prognostic point of view [41,42].

Many researchers indicate that miR-31 is upregulated in OSCC and plays an important role in the progression of cancer [43] and suggest that circulating miR-31-5p may be helpful biomarker in oral cancer diagnosis and a therapeutic target [44]. Moreover, serum miR-31-5p differed significantly between oral cancer patients and healthy subjects, and between pre- and postoperative patients. According to studies by Kavitha [45] overexpression is corelated with a poor clinical outcome. Other authors described low expression of miR-31 -5p in nasopharyngeal cancer tissues which was correlated with the tumor stage [46].

Our study showed higher level of miR-31-5p in EBV positive oropharyngeal cancer patients compared to EBV negative. Moreover, we found that miR-31-5p was higher in moderately and poorly differentiated tumors (G2–G3), in greater tumor dimension (T3–T4) and lymph node involvement (N2-N3). In contrast, the level of miR-let 7a was significantly lower in moderately and poorly differentiated tumor, in extensive tumor dimensions and in lesions with lymph nodes involvement. Luo et al. [47] analyzed the clinical significance of miR-let 7a expression in OSCC by examining the correlation of reduced miR-let 7a levels with clinicopathological features. They found that lower expression of miR-let 7a was significantly correlated only with larger tumor size but not with other clinical features. In addition, the patients with lower miR-let 7a had a poorer prognosis. Moreover, these authors found that downregulation of miR-let 7a promotes cell proliferation, invasion and migration through the miR-let-7a/c-Myc/MAPK/ERK signaling pathway.

Mansouri et al. [48] observing several members of let-7 miR family, described that miR-let 7a inhibit EBV reactivation in EBV-positive NPC cells. They showed the miR-let 7 were upregulated by EBNA1. On the one hand, EBNA1 overexpression increased let-7a expression levels in several cell lines, and on the other hand, EBNA1 silencing reduced let-7a levels. On this basis these authors concluded that EBNA1 upregulate let-7a expression and inhibit EBV reactivation.

Many researchers have analyzed the level of miR-31 in saliva, showing a significant increase in this marker in patients with OSCC [49,50]. Therefore, they postulate that it may be a potential diagnostic biomarker in the early diagnosis and prognosis of head and neck cancer. The aforementioned research on the usefulness of saliva as a diagnostic material concerned NPCs. Therefore, it would be worth checking whether saliva can also be a useful diagnostic material in the oropharyngeal cancer. Although these are preliminary results, they indicate similar miR levels in both the serum and saliva of oropharyngeal cancer patients.

Therefore, in the next stage of the study, we checked also the level of tested miRs in patients’ saliva depending on GTN characteristics. The conducted analysis showed a similar tendency. Therefore, it seems that saliva can be considered a clinical diagnostic material. These observations would, obviously, require confirmation in a larger group of patients.

As mentioned above, EBV establishes a latent infection in which various proteins are synthesized according to latent genes expressed in the host cells. All types of latency express the nuclear antigen EBNA-1 [51,52].

We analyzed also the level of anti EBNA antibodies in the IgG class and additionally EBVCA in the IgG class. The level of both antibodies in EBV-positive patients were significantly higher compared with EBV-negative individuals. From an epidemiological point of view, high titers of both types of antibodies indicate past EBV infection. Understanding the role of the EBV latent genes expressed in oropharyngeal cancers is important to determine the role of viral infection both in the development and tumor progression in this location.

EBV is the first human virus to encode viral miRs [53]. EBV miRs interfere with many biological processes such as cell proliferation, apoptosis, transformation and invasion [54,55]. They can also modulate host defense mechanisms. Viral miRs appear to play a role in regulating lytic and latent infection. Dysregulated miR expressions are closely associated with clinicopathological features and may also be used as independent markers of cancer prognosis [43,56,57].

MiR-BARTs (mostly tested in NPC) contribute to virus latency, cell proliferation and apoptosis, metastasis and tumor recurrence, and participate in the regulation of tumor cell metabolism and immune evasion [58]. Numerous studies have shown that BART miRs expression is low in EBV-infected B cells but high in infected epithelial tissues, suggesting a pathogenic role in the development of epithelial tumors [59,60,61]. Moreover, EBV-miR-BART1 is closely related to clinical stage. They appear to be able regulate cell growth by modulating host gene expression, but this mechanism is not fully understood. In EBV-associated cancers, BARTs are presumed to inhibit most of the latency proteins, reducing the immune response, and only EBNA1 is synthesized [62,63].

The mechanisms discussed above as well as the role of EBV relate to NPC. This mechanism, although not fully understood, may include epigenetic changes (methylation, histone modification) and genetic changes in miR-coding genes [64,65,66]. No studies concerning oropharyngeal cancer linked to EBV have been found in available literature. However, among head and neck cancers in Poland, oropharyngeal cancer dominates. The oropharynx and the nasopharynx are different anatomical regions [2,3]. There are also different risk factors for developing cancer in these areas. Further in-depth research is needed to answer the question of whether the changes at the molecular level in oropharyngeal cancer are similar to those observed in NPC.

The limitation of our research is a relatively small group of patients, especially according to the histological differentiation of the tumor. This made it impossible to accurately determine the difference in miR levels between the moderately and poorly differentiated group. Therefore, G2 and G3 were combined together despite differences from a pathology perspective. Further research is needed to verify the observed trend, as well as to clarify the role of the studied miRs in the development of oropharyngeal cancer. Nevertheless, the obtained results may suggest that the expression of miR-31-5p increases with the progression of histological grade. In contrast, as histologic grade advances miR-let 7a expression decreases. Moreover, the level of tested miRs corelated with clinical stage.

Further research is needed to understand the complex regulatory mechanism of many physiological processes occurring in the EBV-infected host cell. Demonstration of changes in the level of various biomarkers may contribute to the identification of markers of diagnostic and prognostic importance in EBV-related oropharyngeal cancer. In the future, it would be worth checking the level of these miRs in cases of single HPV infection as well as in HPV/EBV co-infection.

In summary, the results presented here demonstrated association of their overexpression and underexpression, in particular stages of the EBV-associated oropharyngeal cancer. In addition, the results of our study may indicate the usefulness of both miRs as diagnostic and prognostic biomakers in EBV related oropharyngeal cancer patients.

## 4. Materials and Methods

### 4.1. Patients

The present study involved 78 patients with a diagnosed and histopathologically confirmed oropharyngeal squamous cell carcinoma (SCC). The study group consisted of 42 patients EBV(+) and 36 patients EBV(−). The patients were hospitalized at Department of Otolaryngology, Head and Neck Cancer, University of Technology and Humanities in Radom, Poland and had not received radiotherapy or chemotherapy before.

Patients were qualified for the study if the p16 immunohistochemical screening test did not show the presence of HPV virus. Subsequently, all HPV negative results were verified by PCR.

The control group consisted of 30 patients of the outpatient clinic in whom cancer was excluded. The control group matched in terms of sociodemographic features. The epidemiological and clinical characteristics of the subjects are presented in Table 1. All patients had the M0 feature. There were no cases of N0 in the study group, because most often patients with oropharyngeal cancer report due to enlarged lymph nodes in the neck (tumor on the neck). The study groups did not differ statistically due to epidemiological and clinical characteristics.

### 4.2. Serum Collection

Venous blood samples from patients and control group were centrifuged at 1500× *g* rpm for 15 min at room temperature and the serum was collected and frozen at −80 °C until analysis.

### 4.3. Saliva Collection

About 5 mL of unstimulated whole saliva was collected. The saliva samples were centrifuged at 1500× *g* rpm at room temperature for 10 min and diluted (1:1) in PBS and frozen at −80 °C until analysis.

### 4.4. Tissue Samples Collection

The tissue samples were collected from all subjects during surgery and frozen at −80 °C until analysis. Tumor, node, metastasis (TNM) classification was determined during primary diagnosis according to the Union for International Cancer Control (UICC) criteria [Non-human Papillomavirus-associated (p16-negative) OPSCC] [16,17,18]. Histological grading was performed according World Health Organization criteria, which divide tumors into three types: well differentiated (G1), moderately differentiated (G2), and poorly differentiated (G3) [67].

#### 4.4.1. DNA Extraction and Detection

Fragments of the freshly frozen tumor tissue (20 mg) collected from all patients with oropharyngeal squamous cell carcinoma (OSCC) were cut and homogenized in a manual homogenizer Omni TH/Omni International/Kennesewa, GA, USA.

DNA was extracted using QIAampDNA Mini Kit (Qiagen, Hilden, Germany) as described in manufacturer’s protocol. Isolated DNA was kept at −20 °C until the test was conducted. To verify the quality of the obtained DNA (presence of inhibitors of Polymerase Chain Reaction), a β-globin assay was performed.

#### 4.4.2. HPV Detection

HPV detection and genotyping was performed using the INNO-LiPA HPV Genotyping Extraassay/Innogenetics/Gent/Belgium. The kit is based on the amplification of a 65 bp fragment from the L1 region of the HPV genome with SPF10 primer set. PCR products are subsequently typed with the reverse hybridization assay.

#### 4.4.3. EBV Detection

EBV DNA was detected using the Gene Proof EBV kit (Brno, Czech Republic) according to manufacturer’s instructions. All samples and a negative control (DNA elution buffer) were analyzed in duplicate. A specific conserved DNA sequence for the EBV nuclear antigen 1 gene (EBNA-1) was amplified using Light Cycle 2.0 Software Version 4.1. (Roche Applied Science System).

#### 4.4.4. miRNA Assay

Serum miRNA levels were determined by the immunoassay enzyme using a commercially available test. MiREIA is a novel, immunoassay-based method of miRNA quantification which involves hybridization of miRNA isolated from a patient sample to complementary biotinylated DNA oligonucleotide probe. These tests were performed according to the manufacturer’s instructions (Biovendor, Brno, Czech Republic). The following kits were used: hsa-miR-31-5-p kit (cat. no.: RDM0044H; limit detection 0.065 amol/μL (6.25–0.195)); hsa-miR-let7a-5p (cat. no.: RDM0023H; limit detection 0.42 amol/μL (40.0–1.25). All samples were tested in triplicate and result was given as arithmetic mean.

#### 4.4.5. Serological Tests

To detect antibody levels serological tests were used with ELISA method. All samples were tested in duplicate. Designed antibodies: anti-VCA IgM (NovaLisa Epstein-Barr Virus VCA IgM/Nova Tec Immunodiagnostica GmbH/Germany/catalog number: EBVM0150), anti-VCA IgG (NovaLisa Epstein-Barr Virus VCA IgG/Nova Tec Immunodiagnostica GmbH/Germany/catalog number: EBVG0150), and anti-EBNA IgG (NovaLisa Epstein-Barr Virus EBNA IgG/Nova Tec Immunodiagnostica GmbH, Dietzenbach, Germany, catalog number: EBVG0580). All tests were performed according to the manufacturer’s instructions.

The NovaTec Epstein-Barr Virus (EBV) IgG-ELISA is intended for the qualitative determination of IgG class antibodies against Epstein-Barr virus. Samples are considered positive if the absorbance value is higher than 10% over the cut-off. The level of antibodies is expressed as NovaTec-Units = NTU.

### 4.5. Statistical Analysis

Results were analyzed using GraphPad Prism software version 9.4.1. Descriptive statistics were used to present the patient’s baseline characteristics. Pearson’s chi-square test was used to investigate the relationship between clinical and demographical parameters. The normal distribution of continuous variables was tested using the Shapiro–Wilk test. The values of the studied parameters were presented as arithmetic means and standard deviations (SD). Medians, maximum and minimum values were also calculated. For variables with an abnormal distribution, the Mann-Whitney U test was used. The correlation between EBNA and EBVCA and both examined miRs level was assessed with Spearman correlation rank test. Statistical significance was defined as *p* < 0.05.

## 5. Conclusions

As other authors emphasize, many different types of cellular MiRs are important in the development and progression of oropharyngeal cancer, including miR-31-p5 and miR-let 7a. Recent studies available in the scientific literature indicate the complexity of the processes occurring in the tumor microenvironment, in which miRs play one of the key roles. Nevertheless, the specific mechanisms of the participation of individual miRs in the process of oncogenesis is not yet fully understood. The interaction of both cellular and viral miRs in different signaling pathways seems most probable. Our data showed that oropharyngeal cancer patients EBV positive are characterized by dysregulation of miR-31-5p and miR-let 7a levels. The obtained results showed a significantly increased level of miR-31-5p and a significantly decreased level of miR-let-7a in the serum of the examined patients. Like other authors, we found that serum and saliva are equally good clinical samples that can be successfully used in diagnostics. In addition, we demonstrated a significant relationship between the serum level of tested miRs and grading and TN classification. Due to the limited number of studies on the role of miR in HNC cancer associated with EBV infection, especially persistent infection, we hope that the presented results will shed new insights into the role of EBV in the promotion of carcinogenesis of this location, including oropharyngeal cancer. Considering the obtained results, further studies are needed to detailed understand the impact of viral miRs on the development and progression EBV related oropharyngeal cancer.

## Figures and Tables

**Table 1:** Epidemiological and clinical characteristics of patients.

		EBV				*p*	Total Patients		Control Group		*p*
		Positive		Negative							
		n	%	n	%		n	%	n	%	
Sex	Female	3	7.14	5	13.89	0.2724	8	10.3	3	10.0	>0.05
	Male	39	92.86	31	86.11		70	89.7	27	90.0	
Age	<50	6	14.29	3	8.33	0.7065	9	11.6	4	13.3	>0.05
	50–69	19	45.24	18	50.0		37	47.4	14	46.7	
	70+	17	40.48	15	41.67		32	41.0	12	40.0	
Place of residence	Urban	29	69.05	21	58.33	0.3254	50	64.1	19	63.3	>0.05
	Rural	13	30.95	15	41.67		28	35.9	11	36.7	
Smoking	Yes	33	78.57	29	80.56	0.8287	62	79.5	23	76.7	>0.05
	No	9	21.43	7	19.44		16	20.5	7	23.3	
Alcohol abuse	Yes	18	42.86	16	44.44	0.8879	34	43.6	14	46.7	>0.05
	No	24	57.14	20	55.56		44	56.4	16	53.3	
G	G1	8	19.05	12	33.33	0.3323	-		-		-
	G2	32	76.19	22	61.11						
	G3	2	4.76	2	5.56						
T	T1-T2	24	57.14	20	96.0	0.1094	-		-		-
	T3-T4	18	42.86	16	4.0						
N	N1	12	28.57	10	27.78	0.9381	-		-		-
	N2-N3	42	71.43	26	72.22						
M	M0	42	100.0	36	100.0	-	-		-		-

Chi^2^ Pearson Test.

## Data Availability

The data presented in this study are available in the article.
